# Satisfaction with care provided by home‐based palliative care service to the cancer patients in Dhaka City of Bangladesh: A cross‐sectional study

**DOI:** 10.1002/hsr2.908

**Published:** 2022-10-29

**Authors:** Jheelam Biswas, Mithila Faruque, Palash Chandra Banik, Nezamuddin Ahmad, Saidur Rahman Mashreky

**Affiliations:** ^1^ Department of Noncommunicable Diseases Bangladesh University of Health Sciences (BUHS) Dhaka Bangladesh; ^2^ Department of Palliative Medicine Bangabandhu Sheikh Mujib Medical University (BSMMU) Dhaka Bangladesh

**Keywords:** Bangladesh, cancer, home‐based palliative care, palliative care, patient satisfaction

## Abstract

**Background and Aims:**

Patient satisfaction is an important quality indicator of health care service. The concept of home‐based palliative care has been recently introduced in Bangladesh, but the patients' satisfaction with this care remained unexplored. This study aimed to assess the satisfaction of the cancer patients receiving this care.

**Methods:**

This cross‐sectional study was conducted among 51 surviving cancer patients above 18 years of age registered under the home‐based care service of the Department of Palliative Medicine, Bangabandhu Sheikh Mujib Medical University, Dhaka, Bangladesh. Data were collected by face‐to‐face interviews using a structured questionnaire based on the FAMCARE P16 questionnaire from February to March 2019. Descriptive analysis was done for the sociodemographic and satisfaction‐related indicators. A correlation matrix was done to see the correlation among the satisfaction indicators.

**Result:**

The majority of the patients (88.2%) were satisfied with the service provided by the home care team. Most (76.5%) of the patients were women, and the mean age was 56.25 ± 14.8 years. The median duration of getting home‐based care was 4 months. Main satisfaction indicators were—assessment of physical symptoms (70.6%), providing information about pain management (70.6%), the inclusion of the family in decision making (76.5%), coordination of care between the members of the home care team (84.3%) and availability of doctors, nurses and palliative care assistants (74.5%). A high correlation was observed between satisfaction regarding the care of physical symptoms and provision of information (*R* = 0.814, *p* < 0.001). Also, satisfaction regarding the provision of information and support provided to the family is highly correlated (*R* = 0.722, *p* < 0.001).

**Conclusion:**

Despite the limitations, the overall satisfaction level of the patients regarding home‐based palliative care services in Bangladesh is very high. Home‐based palliative can be a solution to provide palliative care to patients who are unable to access institution‐based care and improve their quality of life.

## 1 BACKGROUND

Palliative care is a holistic approach that focuses on improving the quality of life of patients with life‐limiting illness and their families by assessment and prevention of physical, psychosocial, and spiritual sufferings.^1^ In the last few decades, there is an enormous growth in the field of palliative care, and it has been integrated into the mainstream health care system in many countries.^2^, ^3^, ^4^, ^5^ Still, about 40 million people worldwide are in the need of palliative care, 34% of them are diagnosed with cancer of different stages, but only about 14% of them are currently receiving this care.^6^

The diagnosis of cancer itself and its treatment‐related side effects give rise to various physical, psychosocial, and spiritual sufferings.^7^ It has been evidenced that palliative care helps this patient and their families to deal with these problems.^1^ Among different palliative care delivery system models, the cost‐effectiveness and higher patient satisfaction reported in home‐based palliative care services increased the popularity of this service around the world.^8^

Although in many countries, palliative care is an essential part of the health care system, in many lower‐middle and lower‐income countries, the concept of palliative care is still new. In Bangladesh, there are some isolated initiatives of providing home‐based palliative care have been taken. Approximately 0.6 million patients need palliative care in Bangladesh, but less than 4000 people have received this care until now.^9^, ^10^ Along with institutional care, a few private and autonomous institutes are providing home‐based palliative care in a small scale. But most of these services are confined within the capital city. Due to a lack of proper recordkeeping and collaboration, the extent of these services remained unexplored. The Department of Palliative Medicine of Bangabandhu Sheikh Mujib Medical University (BSMMU) has taken a pioneering role, and has been delivering home‐based palliative care since 2008 in an organized way.

Patient satisfaction is one of the main indicators in assessing the quality of the service provided by the home‐care team. However, there is no such study done to assess the satisfaction level of cancer patients receiving this service. This study assessed the satisfaction with care in the cancer patients receiving home‐based palliative care in Bangladesh.

## 2 METHODS

### 2.1 Study design and setting

This cross‐sectional study was conducted amongst all surviving cancer patients currently registered under the Department of Palliative Medicine, BSMMU, Shahbag, Dhaka, Data collection was carried out in February and March 2019.

### 2.2 Home‐based palliative care

The home‐based palliative care service is provided by the Department of Palliative Medicine, BSMMU to the registered patients living within 20 km radius of the university. The home care team consists of one doctor, two to three nurses, and five trained palliative care assistants (PCAs). They work 5 days per week. Patients who are unable to visit hospital due to physical condition, distance, financial constraints, or lack of caregivers are considered to be eligible for this service irrespective of their disease stage. This service includes wound care, adjusting medications, general physical and mental care and follow‐up, interaction with the caregivers to give temporary respite from their caregiving duties, as well as bereavement support. The PCA includes specially trained individuals who do the initial visits. They are involved in minor wound care, general physical care, helping the family caregivers, and listening to the patients' and their caregivers' problems. Doctors and nurses visit each patient once a week. They are involved in major wound care, adjustment of medication, and communication with the patients and their family members about which the PCAs cannot elaborate. The visits are need based. Usually, every patient gets two to three visits per month, although extra visits are given based on patients' condition and caregiver demand.

Satisfaction is the fulfillment of the individual needs and expectations of the patients and their families, and is assessed by a two‐way communication between the receivers of the care and the provider of the care.^11^ It depends on various factors like the aspects of service provided by the team, medical and backup care, as well as various organizational components co‐coordinating with each other.^12^ Evaluation of patient satisfaction is necessary to assess the quality of the service and planning for necessary intervention. Improving patient satisfaction leads to improving the quality of the service.

### 2.3 Sample criteria

All the surviving cancer patients who registered under this service up to February 2019, above 18 years of age, willing to participate, and got at least three visits from the home care team were included in the study. Those who were delirious, disoriented, or unable to communicate were excluded. Those caregivers (paid or family members) who take care of the patients at least 5 days per week are included in the study. Occasional caregivers were excluded.

### 2.4 Sample size

According to the Center of Palliative Care (CPC) database up to February 2019, the number of registered cancer patients receiving home‐based palliative care was 60. During data collection three patients died, four patients were not eligible for the study due to delirium, and three patients refused to give informed consent. So the final sample size of the study was 51.

### 2.5 Instruments

The questionnaire had two major parts. The first part contained the sociodemographic, disease, treatment, and primary caregiver‐related information collected from the hospital record.

The second part of the questionnaire contained questions from the FAMCARE P‐16 questionnaire developed by Lo et al.^13^ which was used to assess the satisfaction level of the study subjects. This instrument had four indicators—care of physical symptoms (items 1, 2, 5, 7, 8), providing information (items 2, 4, 6, 9, 10), family support (items 14, 15, 16), access to care (items 11, 12, 13). This part of the questionnaire was translated in Bangla based on the methodology developed Eremenco et al.^14^ At first two native Bangla speakers translated the questionnaire into Bangla, and then the synthesis of these two translations was done by the experts from the Bangladesh University of Health Sciences. Again, the back‐translation of the synthesized Bangla version was done by an English language expert. After comparing the translated and back‐translated versions, necessary corrections were made by a panel of linguistic experts. The corrected version was tested among 10 cancer patients (10% of the total sample size) admitted to both the palliative medicine and oncology department of BSMMU for linguistic and content validation. Necessary wording or sentence structure changes were made based on the patients' responses. Then the revised version was again reviewed by a panel of linguistic experts. After completing the linguistic validation, the questionnaire was finalized.

### 2.6 Data collection procedure

Initial data were collected from the hospital case sheets (sociodemographic information, diagnosis and treatment history, caregiver‐related information).

The investigators accompanied the home care team to the patients' home, and the interviews were conducted in their presence. Informed consent was obtained from both the patients and their primary caregivers. Mini Mental State Examination (MMSE) was done to determine the consent‐giving capacity of the patient. The consent was obtained either in written or verbal form depending on the patients' condition.

The patients and the caregivers were recruited in pair and were interviewed together. The duration of each interview was 30 minutes to 1 hour. Very frail patients were given multiple visits to complete an interview.

### 2.7 Data analysis

All data were analyzed by SPSS version 22.0 after editing and logical checking.

Categorical variables such as sex, education, marital and occupational status, knowledge and belief about the disease prognosis, treatment, and side effects, the relationship of the primary caregiver with the patient were reported as frequency and percentage. Continuous variables such as age, monthly family income, duration of getting home‐based palliative care were presented in mean, SD, and median as appropriate. Number of satisfied patients with each component was presented in frequency and percentage.

The level of satisfaction was presented in three categories based on mean and SD. The value below lower limit of mean − 1  SD was categorized as not satisfied, the range between upper and lower limit of mean ± 1 SD was categorized as satisfied and the value above mean + 1 SD was categorized as very satisfied.

Correlation matrix was done to see the correlation among the satisfaction indicators. *p* < 0.05 was considered as significant.

## 3 RESULTS

The majority (76.5%) of the patients were women, and the mean age was 56.25 ± 14.8 years. More than half (58.8%) of the patients were married and lived with their partners. Almost 97% of the patients had family members as their primary caregivers, mostly their children (53.2%) or spouses (29.8%), and 57.6% of the primary caregivers were women. Common sites of the primary cancer were breast (39.2%), genitourinary system (23.5%), and gastrointestinal tract (17.6%). More than half (55.8%) of the patients had metastasis at the time of referral to palliative care, and 80% of them were currently only on palliative management. The median duration of receiving home‐based palliative care of the patients was four months (ranging from 6 days to 1 year) (Table 1).

**Table 1:** Sociodemographic characteristics of the patients and primary caregivers

Variables	*n* (%)	95% CI	
		Lower bound	Upper bound
*Sociodemographic characteristics of the patients (n = 51)*			
Sex			
Men	12 (23.5)	11.9	35.1
Women	39 (76.5)	64.9	88.1
Age, years			
Mean ± SD	56.25 ± 14.8		
<45	13 (25.5)	13.5	37.5
45–65	26 (51.0)	37.3	64.7
>65	12 (23.5)	11.9	35.1
Marital status			
Single (unmarried/divorced/widow)	21 (41.2)	27.7	54.7
Married	30 (58.8)	45.3	72.3
Educational status			
Illiterate	6 (11.8)	2.9	20.7
Primary	16 (31.4)	18.7	44.1
Up to higher secondary	18 (35.3)	22.2	48.4
Graduate or above	11 (21.6)	10.3	32.9
Primary sites of cancer			
Gastrointestinal system	9 (17.6)	7.1	28.1
Genitourinary system	12 (23.5)	11.9	35.1
Breast	20 (39.2)	25.8	52.6
Others	10 (5.6)	0.0	11.9
Staging of cancer at referral			
Up to stage III	12 (23.1)	11.5	34.7
Stage IV	25 (48.1)	34.4	61.8
Unknown	15 (28.8)	16.4	41.2
Duration of getting home‐based palliative care (months)			
<1	18 (35.3)	22.2	48.4
1–6	15 (29.4)	16.9	41.9
>6	18 (35.3)	22.2	48.4
Median duration (months)	4		
*Characteristics of primary caregivers (n = 47)* a			
Age, years			
Mean ± SD	42.3 ± 16.45		
<31	15 (31.9)	13.3	18.6
31–50	20 (42.6)	28.5	56.7
>50	12 (25.5)	13.0	38.0
Sex			
Men	20 (42.5)	28.4	56.6
Women	27 (57.4)	43.3	71.5
Relationship with the patients			
Spouse	14 (29.8)	16.7	42.9
Children	25 (53.2)	38.9	67.5
Others	8 (17.0)	6.3	27.7

Abbreviation: CI, confidence interval.

^a^ Four participants had no caregiver.

The majority of the patients were very satisfied with the assessment of physical symptoms (70.6%), providing information about pain management (70.6%), the inclusion of the family in decision making (76.5%), coordination of care between the members the home care team (84.3%) and availability of doctors, nurses and PCAs in the time of need (74.5%) (Table 2).

**Table 2:** Indicators of patient satisfaction (*n* = 51)

Variables	No of respondents, *n* (%)			
	VD	UD	S	VS
Care of physical symptoms				
Doctor's attention to symptom description	0	0	10 (19.6)	41 (80.4)
Assessing the symptoms	0	0	15 (29.4)	36 (70.6)
Treatment of the symptoms	2 (3.9)	0	28 (54.9)	21 (41.2)
Tests performed	0	5 (9.8)	30 (58.8)	16 (31.4)
Follow up	0	1 (2)	31 (60.8)	19 (37.3)
Providing information				
Pain management	1 (2)	2 (3.9)	12 (23.5)	36 (70.6)
Treatment side effects	1 (2)	3 (5.9)	29 (56.9)	18 (35.3)
About tests and reports	0	9 (17.6)	28 (54.9)	14 (27.5)
About prognosis	1 (2)	4 (7.8)	20 (39.2)	26 (51.1)
Answering the question asked			13 (25.5)	38 (74.5)
Support to the family				
Inclusion of family in decision making	0	1 (2)	11 (21.6)	39 (76.5)
Coordination of care	0	1 (2)	7 (13.7)	43 (84.3)
Availability of doctors to the family	0	1 (2.0)	21 (41.2)	29 (56.9)
Access to the care				
Referral to specialist		43 (84.3)	3 (5.9)	5 (9.8)
Availability of doctors			13 (25.5)	38 (74.5)
Availability of nurses and PCA	1 (2.0)	2 (3.9)	10 (19.6)	38 (74.5)

*Note*: Five‐point Likert's scale used, unsatisfied scale is not shown in the table as no one reported it.

Abbreviations: PCA, palliative care assistant; S, satisfied; UD, undetermined; VD, very dissatisfied; VS, very satisfied.

**Figure 1:**
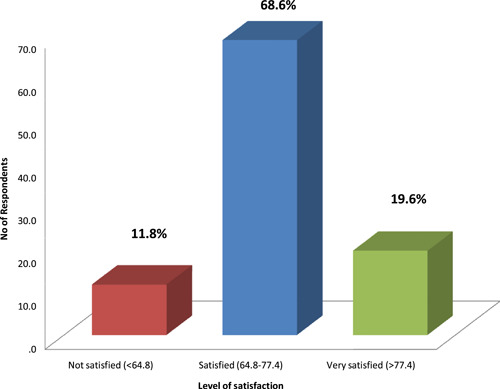
Level of satisfaction (categorization done based on mean ± 1 SD (71.1 ± 6.3)

The majority of the patients (88.2%) were satisfied with the service provided by the home care team (Figure 1).

High correlation was observed between satisfaction regarding the care of physical symptoms and satisfaction regarding the provision of information (*R* = 0.814, *p* < 0.001), followed by satisfaction regarding the support given to the family (*R* = 0.759, *p* < 0.001). Also, satisfaction regarding the provision of information and satisfaction regarding the support given to the family were highly and significantly correlated (*R* = 0.722, *p* < 0.001) (Table 3).

**Table 3:** Correlation matrix of satisfaction indicators

	*R* value			
Satisfaction Indicators	Care of physical symptoms	Providing information	Support to the family	Access to the care
Care of physical symptoms	1.000	0.814	0.759	0.644
Providing Information		1.000	0.722	0.635
Support to the family			1.000	0.581
Access to the care				1.000

*Note*: *p* value represents highly significant (*p* < 0.001) between the scales.

## 4 DISCUSSION

Home‐based palliative care has been introduced recently in Bangladesh. This is the first study in Bangladesh assessing the satisfaction with the care of the patients' receiving such care.

Measuring patients' satisfaction is important for evaluating the outcome of the care provided by the home care team. It gives valuable information about the patient's experience with the service, measures their compliance with the treatment, identifies the underlying weaknesses, and evaluates the performance of the home care team.^15^, ^16^ In our study, the majority (88.2%) of the patients are satisfied with the service provided by the home care team. This finding is almost close to the satisfaction level (93%–96%) of the countries like the United States with well‐developed home‐based palliative care delivery systems.^17^ This satisfaction level is also similar to the satisfaction level of the patients from India receiving such care, where isolated home‐based palliative service is present. In both scenarios, the satisfaction level of the patients with the care provided by the home care team is very high.^18^ This scenario shows a ray of hope in a country where home‐based palliative care is not a well‐known concept and mostly depends on patronage of a few institutions.

The majority (80.4%) of the patients in this study are very satisfied with the attention given by the home care team members during the assessment of their sufferings. It indicates better communication between the home care team and the patients, where more than half (61%) of the patients attending government hospitals of Bangladesh show dissatisfaction about the health care providers' lack of attention to their symptom description.^19^ In this study, patients receiving home‐based palliative care are also very satisfied with the information provided by the home care team about their disease and treatment. Satisfaction regarding the care of physical symptoms and satisfaction regarding the provision of information are also found to be highly correlated, which means giving proper information about the disease and treatment improves the patients' satisfaction regarding physical symptom care. This finding is on par with the performance of the home care team of Kerala, India, which is considered as a model for the developing home‐based palliative care service like ours.^18^

The majority of the patients in our study are very satisfied with the inclusion of family members in decision making (76.5%) and availability of the doctors and nurses in need (75.5%), in contrast to earlier studies where the main dissatisfaction arises from the long waiting time for the doctors and nurses in hospitals.^19^ Also the inclusion of family members in decision making are mostly ignored in most hospital setting in Bangladesh, so the family members do not feel connected with the health care team.^20^ We have also observed that satisfaction regarding the care of physical symptoms and provision of information are highly correlated with satisfaction regarding the support provided to the family members. It indicates that to achieve the patients' satisfaction in other areas, support to their family members must be given. The approach to the patients' families by the home care team can be used as an example to the rest of health workers in Bangladesh on patients' family health care team relationship. Among many of the barriers in accessing health services in Bangladesh, one of the major barriers are long travel distances to facilities, and long waiting times once facilities were reached.^21^ High satisfaction level with home‐based palliative care indicates that it can be a solution to provide necessary care to the patients in need and improve their satisfaction level.

One major limitation of this study is that this study was conducted on the home‐based palliative care service provided by a single institution, so the findings of the study cannot be generalized. Also the greater picture of home‐based palliative care in Bangladesh is not reflected in this study. Due to the cross‐sectional nature of this study, the perception of satisfaction among the patients cannot be measured over time.

## 5 CONCLUSION

Patient satisfaction is one of the important quality indicators of health care service. Despite the limitations, overall satisfaction on the care provided by the home care team is high. As home‐based palliative care continues to develop, further research is needed to evaluate the satisfaction level of the patients over time.

## AUTHOR CONTRIBUTIONS

**Jheelam Biswas**: Conceptualization; data curation; formal analysis; investigation; methodology; project administration; resources; software; validation; visualization; writing–original draft; writing–review and editing. **Mithila Faruque**: Conceptualization; formal analysis; project administration; software; supervision; validation; writing–original draft; writing–review and editing. **Palash Chandra Banik**: Conceptualization; data curation; formal analysis; methodology; project administration; software; supervision; validation; writing–original draft; writing–review and editing. **Nezamuddin Ahmad**: Funding acquisition; resources; supervision; validation; writing–original draft; writing–review and editing. **Saidur Rahman Mashreky**: Conceptualization; formal analysis; methodology; supervision; validation; visualization; writing–original draft; writing–review and editing.

## CONFLICT OF INTEREST

The authors declare no conflict of interest.

## ETHICS STATEMENT

Ethical approval for both the research and consent procedure (Approval no: BUHS/BIO/EA/18/158, date:18/10/2018) was obtained from the Ethical Review Committee, Bangladesh University of Health Sciences (BUHS), and permission for data collection was obtained from the Department of Palliative Medicine, BSMMU. The written informed consent was taken from all the eligible patients and their primary caregivers. As they were terminally ill patients, their health conditions were considered during data collection.

## TRANSPARENCY STATEMENT

The lead author Jheelam Biswas affirms that this manuscript is an honest, accurate, and transparent account of the study being reported; that no important aspects of the study have been omitted; and that any discrepancies from the study as planned (and, if relevant, registered) have been explained.

## Supporting information

Supporting information.

Supporting information.

## Data Availability

The data that support the findings of this study are openly available in Mendely data doi:  10.17632/d4jzhzxjzj.1. All authors have read and approved the final version of the manuscript and the lead author Dr. Jheelam Biswas has full access to all of the data in this study and takes complete responsibility for the integrity of the data and the accuracy of the data analysis.
