# Supplementary material for: Satisfaction with care provided by home‐based palliative care service to the cancer patients in Dhaka City of Bangladesh: A cross‐sectional study
Source: Health Sci Rep. 2022 Oct 29;5(6):e908. doi: 10.1002/hsr2.908 (PMC9617647; doi:10.1002/hsr2.908)
Supplement: Supplementary file 1 — Supporting information. [file HSR2-5-e908-s001.docx]

**Assessing level of satisfaction [FAMCARE P-16 ]**

**Instructions:**

Please answer the questions below indicating how satisfied you are with the care received: very satisfied (VS), satisfied (S), undecided (U), dissatisfied (D), very dissatisfied (VD). Please circle the letters below that best match your experience.

**How satisfied are you with:**

| **Sl** | **Questions** | **VD** | **D** | **U** | **S** | **VS** |
| --- | --- | --- | --- | --- | --- | --- |
| 1 | Doctor’s attention to your description of symptoms | 1 | 2 | 3 | 4 | 5 |
| 2 | How thoroughly the doctor assesses your symptoms | 1 | 2 | 3 | 4 | 5 |
| 3 | Information given about how to manage pain | 1 | 2 | 3 | 4 | 5 |
| 4 | Information given about side effects | 1 | 2 | 3 | 4 | 5 |
| 5 | Speed with which symptoms are treated | 1 | 2 | 3 | 4 | 5 |
| 6 | Information given about your tests | 1 | 2 | 3 | 4 | 5 |
| 7 | The way tests and treatments are performed | 1 | 2 | 3 | 4 | 5 |
| 8 | The way tests and treatments are followed up by the doctor | 1 | 2 | 3 | 4 | 5 |
| 9 | Information provided about your prognosis | 1 | 2 | 3 | 4 | 5 |
| 10 | Answers from health professionals | 1 | 2 | 3 | 4 | 5 |
| 11 | Referrals to specialists | 1 | 2 | 3 | 4 | 5 |
| 12 | The availability of doctors to answer your questions | 1 | 2 | 3 | 4 | 5 |
| 13 | The availability of nurses to answer your questions | 1 | 2 | 3 | 4 | 5 |
| 14 | The way the family is included in treatment and care decisions | 1 | 2 | 3 | 4 | 5 |
| 15 | Coordination of care | 1 | 2 | 3 | 4 | 5 |
| 16 | The availability of the doctor to your family | 1 | 2 | 3 | 4 | 5 |
