# Supplementary material for: Satisfaction with care provided by home‐based palliative care service to the cancer patients in Dhaka City of Bangladesh: A cross‐sectional study
Source: Health Sci Rep. 2022 Oct 29;5(6):e908. doi: 10.1002/hsr2.908 (PMC9617647; doi:10.1002/hsr2.908)
Supplement: Supplementary file 2 — Supporting information. [file HSR2-5-e908-s002.docx]

|  | **প্রশ্ন** | **ভীষণ অসন্তুষ্ট** | **অসন্তুষ্ট** | **নিশ্চিতভাবে বলতে পারছি না** | **সন্তুষ্ট** | **খুবই সন্তুষ্ট** |
| --- | --- | --- | --- | --- | --- | --- |
| 1 | আপনার সমস্যাগুলির বর্ণনা ডাক্তার যেভাবে মনোযোগ দিয়ে শুনেছেন, তা নিয়ে কি আপনি সন্তুষ্ট | ১ | ২ | ৩ | ৪ | ৫ |
| 2 | ডাক্তার যতটুকু গভীরভাবে আপনার সমস্যাগুলি মূল্যায়ন করেছেন, তা নিয়ে কি আপনি সন্তুষ্ট | ১ | ২ | ৩ | ৪ | ৫ |
| 3 | কিভাবে ব্যথা নিয়ন্ত্রন করতে হয় সেই সম্পর্কে আপনাকে যে তথ্য দেওয়া হয়েছে, তা নিয়ে কি আপনি সন্তুষ্ট | ১ | ২ | ৩ | ৪ | ৫ |
| 4 | চিকিৎসার পার্শ্বপ্রতিক্রিয়া সম্পর্কে আপনাকে যে তথ্য দেওয়া হয়েছে, তা নিয়ে কি আপনি সন্তুষ্ট | ১ | ২ | ৩ | ৪ | ৫ |
| 5 | যেভাবে দ্রুততার সাথে আপনার সমস্যাগুলির চিকিত্সা প্রদান করা হয়েছে, তা নিয়ে কি আপনি সন্তুষ্ট | ১ | ২ | ৩ | ৪ | ৫ |
| 6 | রোগ নির্ণয় এবং চিকিৎসার জন্য যে সকল পরীক্ষানীরিক্ষা প্রয়োজন, তা সম্পর্কে যে সকল তথ্য আপনাকে দেওয়া হয়েছে, তা নিয়ে কি আপনি সন্তুষ্ট | ১ | ২ | ৩ | ৪ | ৫ |
| 7 | যে সকল পরীক্ষানীরিক্ষা এবং চিকিত্সা আপনাকে দেওয়া হয়েছে, তা নিয়ে কি আপনি সন্তুষ্ট | ১ | ২ | ৩ | ৪ | ৫ |
| 8 | ডাক্তার যেভাবে আপনার পরীক্ষানীরিক্ষার ফলাফল এবং প্রদানকৃত চিকিত্সাসমূহকে পুনরায় মূল্যায়ন করেছেন, তা নিয়ে কি আপনি সন্তুষ্ট | ১ | ২ | ৩ | ৪ | ৫ |
| 9 | আপনার রোগের পরিণতি সম্পর্কে আপনাকে যে সকল তথ্য প্রদান করা হয়েছে, তা নিয়ে কি আপনি সন্তুষ্ট | ১ | ২ | ৩ | ৪ | ৫ |
| 10 | ডাক্তার, নার্স এবং অন্যদের কাছে আপনার রোগ এবং চিকিৎসা সম্পর্কিত প্রশ্ন করে যে সকল উত্তর পেয়েছেন, তা নিয়ে কি আপনি সন্তুষ্ট | ১ | ২ | ৩ | ৪ | ৫ |
| 11 | বিশেষজ্ঞ চিকিৎসকদের কাছে থেকে মতামত নেওয়া এবং তাঁদের কাছে পাঠানোর ব্যাপারে যে সকল ব্যবস্থা নেওয়া হয়েছে, তা নিয়ে কি আপনি সন্তুষ্ট | ১ | ২ | ৩ | ৪ | ৫ |
| 12 | আপনার প্রশ্নের উত্তর দেবার জন্য ডাক্তারদেরকে যেভাবে সহজে পাওয়া গিয়েছে, তা নিয়ে কি আপনি সন্তুষ্ট | ১ | ২ | ৩ | ৪ | ৫ |
| 13 | আপনার প্রশ্নের উত্তর দেবার জন্য নার্সদেরকে যেভাবে সহজে পাওয়া গিয়েছে, তা নিয়ে কি আপনি সন্তুষ্ট | ১ | ২ | ৩ | ৪ | ৫ |
| 14 | আপনার চিকিত্সা এবং যত্ন সম্পর্কিত ব্যাপারে সিদ্ধান্ত গ্রহণে আপনার পরিবারকে যেভাবে অন্তর্ভুক্ত করা হয়েছ, তা নিয়ে কি আপনি সন্তুষ্ট | ১ | ২ | ৩ | ৪ | ৫ |
| 15 | ডাক্তার, নার্স এবং অন্যান্যদের প্রদানকৃত যত্নের মাঝে যে সমন্বয় দেখতে পেয়েছেন, তা নিয়ে কি আপনি সন্তুষ্ট | ১ | ২ | ৩ | ৪ | ৫ |
| 16 | প্রয়োজনে আপনার পরিবারের পাশে দাঁড়ানোর জন্য ডাক্তার/ নার্সদেরকে যেভাবে সহজে পাওয়া গিয়েছে, তা নিয়ে কি আপনি সন্তুষ্ট | ১ | ২ | ৩ | ৪ | ৫ |
